# Temperature-Dependent Changes in Physicochemical Properties and Selected Bioactive Compounds During Controlled Fermentation of Cocoa Beans (*Theobroma cacao*)

**DOI:** 10.3390/foods15193473

**Published:** 2026-09-28

**Authors:** Theerapong Kammoon, Sathian Boonkum, Patcharee Pattanagul, Pratchaya Tipduangta, Supavej Maniyom

**Affiliations:** 1 Faculty of Agro-Industry, Chiang Mai University, Chiang Mai 50100, Thailand; kammoont@gmail.com (T.K.); sathian.b@cmu.ac.th (S.B.); mrsupavej@gmail.com (P.P.); 2 Audiva Thai Cacao Co., Ltd., Chiang Mai 50230, Thailand; 3 Department of Pharmaceutical Sciences, Faculty of Pharmacy, Chiang Mai University, Chiang Mai 50200, Thailand; pratchaya.t@cmu.ac.th

**Keywords:** cocoa fermentation, controlled temperature, physicochemical properties, metabolite dynamics, theobromine, catechin

## Abstract

Cocoa bean (*Theobroma cacao* L.) fermentation is an important postharvest process that influences the physicochemical characteristics and quality of cocoa beans. This study investigated the effects of controlled fermentation temperatures (15, 25, 35, and 45 °C) on physicochemical properties and metabolite dynamics. Beans were fermented for 8 days without turning during the first 2 days, followed by daily turning and drying at 50 °C for 3 days. Fermentation temperature significantly affected physicochemical properties, metabolite profiles, and quality-related characteristics of dried cocoa beans. Fermentation at 15 °C showed characteristics of less extensive fermentation, including the highest yield (47.17%), water activity (0.57), reducing sugar (17.84 mg/g), and total phenolic compounds (2.39 mg/g), together with the lowest total titratable acidity (0.26%). In contrast, fermentation at 45 °C resulted in the lowest yield (37.67%), total soluble solids (2.23 °Brix), moisture content (4.11%), water activity (0.46), reducing sugar (3.24 mg/g), and total phenolic compounds (0.36 mg/g), while producing the highest total titratable acidity (0.39%). Theobromine content remained unchanged (3.38–3.87 mg/g), whereas catechin increased slightly (0.03–0.10 mg/g) with increasing temperature. These findings support temperature-controlled fermentation for improving postharvest quality control and value addition in cocoa production.

## 1. Introduction

Cocoa bean fermentation is an important post-harvest process that plays a crucial role in the development of cocoa bean quality and the formation of flavor precursors in chocolate products. Several factors influence cocoa bean fermentation, including pod ripeness, cocoa variety, fermentation batch size, fermentation duration, turning frequency, and process conditions such as temperature and pH, all of which affect the physicochemical changes in the beans and the development of flavor precursors [1,2,3,4,5]. In addition, cocoa fermentation involves the coordinated activity of several groups of microorganisms, including yeasts, lactic acid bacteria, acetic acid bacteria, and molds [6,7,8]. The process begins with the degradation of sugars and nutrients present in the cocoa pulp, leading to the production of various metabolites such as ethanol, organic acids, and precursor compounds [9,10]. These processes also result in significant physical and chemical changes in cocoa beans such as reducing sugar content, total acidity, phenolic compounds, bioactive compounds, as well as color attributes [11,12,13].

Temperature is considered one of the most important factors affecting microbial growth and metabolic activity during cocoa fermentation [6,14]. Typically, the fermentation temperature initially starts close to the ambient temperature of approximately 25–30 °C. Subsequently, microbial metabolic activities generate heat, causing the temperature to increase to approximately 35–45 °C during the fermentation period [15,16,17]. Changes in fermentation temperature can influence the rate of substrate utilization, such as sugars, and the production of metabolites including ethanol and organic acids, which play important roles in the overall quality of cocoa beans [1,18,19]. In addition, temperature also affects the transformation of phenolic compounds, which are associated with antioxidant properties and chemical attributes of cocoa products [11,20,21].

Although previous studies have reported that the temperature range of approximately 25–50 °C is favorable for microbial growth during cocoa fermentation, variations in fermentation temperature may influence the kinetics of the fermentation process as well as changes in the physical and chemical quality of the resulting cocoa beans [15,22]. Cocoa fermentation is predominantly conducted as a spontaneous process at the farm or local-cooperative level, and differences in agricultural and operational practices can result in considerable variation in fermentation performance and final bean quality [16]. Recent reviews have emphasized that postharvest handling, particularly fermentation, drying, and storage, plays a critical role in determining cocoa bean quality and that improved control of microbial activity and fermentation conditions is needed to achieve more consistent quality [23]. Moreover, systematic evidence indicates that spontaneous cocoa fermentations are more variable than previously recognized, highlighting the need to better understand the key fermentation parameters that can be manipulated to improve process consistency [24]. For smallholder cocoa production systems, practical and resource-efficient approaches to postharvest management are therefore particularly important, as process control needs to be achievable without requiring complex or costly technologies. Among the controllable fermentation parameters, temperature is a critical factor influencing physicochemical and biochemical changes that contribute to cocoa bean quality. Therefore, investigating the effects of different fermentation temperatures is essential not only for understanding the mechanisms underlying cocoa fermentation but also for identifying temperature conditions that may support more consistent and controllable cocoa bean processing for production purposes [22,25,26].

The objective of this study was to investigate the effects of different fermentation temperatures (15, 25, 35, and 45 °C) on the kinetic changes and quality of cocoa beans during the fermentation process, with the aim of identifying a resource-efficient fermentation strategy that supports sustainable cocoa processing. The cocoa beans were fermented without turning during the first two days, followed by daily turning until the completion of the eight-day fermentation period. Samples were collected at two-day intervals throughout fermentation to monitor changes in selected physicochemical properties. After 8 days of fermentation, the beans were dried and evaluated for final quality attributes. The study evaluated the changes in physical and chemical properties, including moisture content, reducing sugar content, total acidity, phenolic compounds, bioactive retention, and color attributes of the dried cocoa beans. The results obtained from this study are expected to provide useful information for determining appropriate fermentation conditions to obtain high-quality cocoa beans, offering a practical postharvest quality control approach that can support value addition within smallholder cocoa production systems.

## 2. Materials and Methods

### 2.1. Materials

Fresh cocoa pods (*Theobroma cacao* L.) of the Forastero ‘Chumphon 1 Hybrid’ variety, at 40–80% ripeness, were obtained from One Farm in Saraphi District, Chiang Mai Province, Thailand. The pods were stored for 48 h before being manually split open, after which 5 kg of beans per batch were collected. The fresh beans were drained for 3 h to remove excess pulp liquid before being transferred into 9 L insulated ice coolers (diameter × height: 27 × 35.5 cm; model M9JB, Picnic, Thailand). The bean mass in each cooler was covered with banana leaves to simulate traditional fermentation conditions.

A total of 12 fermentation containers were prepared and incubated at four controlled temperatures (15, 25, 35, and 45 °C), with three independent replicates for each temperature treatment. During the first two days, the cocoa beans were fermented under anaerobic conditions without turning. From day 3 to day 8, the bean mass was turned daily to promote aerobic fermentation.

Samples of fermenting cocoa beans were collected every two days throughout the fermentation period for physicochemical analyses and kinetic characteristics. At the end of the 8-day fermentation process, fermented beans were dried in hot-air oven (a 6 tray-hot-air oven, FNB Machinery and Solution, Bangkok, Thailand) at 50 °C for 3 days and subsequently analyzed to determine their physicochemical quality.

### 2.2. Methods

#### 2.2.1. Measurement of Temperature, Color Values, Total Soluble Solids, pH, and Water Activity

The temperature of the fermenting cocoa beans was monitored using a portable 3-in-1 soil tester (MUCIAKIE Soil Tester, Shanghai Yuqiu Co., Ltd., Shanghai, China). Temperature measurements were recorded every 2 days by averaging readings obtained from three different points within the fermentation bucket (top, middle, and bottom).

The color of cocoa beans was determined using the Hunter L*, a*, and b* color system with colorimeter (Konica Minolta CR-400 colorimeter, Tokyo, Japan). Total soluble solids (TSS) were measured using a hand refractometer (RHB-10ATC, MLOPTIC (Thailand) Co., Ltd., Chonburi, Thailand). The pH value was determined using a pH meter (C830, Consort multi-parameter analyzer, Turnhout, Belgium). The water activity (a_w_) was measured by directly using a water activity meter (Pre Aqua Lab Water Analyzer, Pullman, WA, USA).

#### 2.2.2. Determination of Moisture Content

Moisture content was determined according to the AOAC International Official Method 925.10 [27]. Approximately 5.0 g of ground fermented and dried cocoa bean samples were accurately weighed into a pre-dried and pre-weighed moisture dish. The samples were dried in a hot-air oven at 105 ± 2 °C until a constant weight was achieved (approximately 3–5 h). After drying, the moisture dishes were transferred to a desiccator and allowed to cool to room temperature before being weighed.

Moisture content was calculated based on the weight loss during drying and expressed as a percentage of the initial sample weight using the following equation:

$$\mathrm{Moisture}\;\mathrm{content}\;\left(\%\right)=\frac{W_{1}-W_{2}}{W_{1}}\times 100$$

where:

- *W*_1_ = initial weight of the sample (g);
- *W*_2_ = weight of the dried sample (g).

All measurements were performed in triplicate, and the results were expressed as mean ± standard deviation.

#### 2.2.3. Determination of Reducing Sugar

The concentrations of reducing sugars in the fermented and dried cocoa bean samples were determined using the 3,5-dinitrosalicylic acid (DNS) method, as originally described by Miller [28]. In this method, 1.0 mL of appropriately diluted sample or glucose standard solution was mixed with 1.0 mL of DNS reagent in a test tube. The mixture was heated in a boiling water bath for 5 min to allow color development, then immediately cooled to room temperature under running tap water. After cooling, 8.0 mL of distilled water was added to each tube to dilute the solution. The absorbance was measured at 540 nm using a UV–Vis spectrophotometer. A calibration curve was prepared using standard glucose solutions, and the concentration of reducing sugars in the sample was expressed as milligrams of glucose equivalents per milliliter or per gram of sample.

$$\mathrm{Reducing}\;\mathrm{sugar}\;(\mathrm{mg}/g)=\frac{C\times V\times \mathrm{DF}}{W}\;$$

where *C* is the glucose concentration determined from the standard calibration curve (mg/mL), *V* is the total volume of the sample extract (mL), *DF* is the dilution factor, and *W* is the dry weight of the sample (g).

#### 2.2.4. Determination of Total Phenolic Content

Total phenolic content (TPC) was determined using the Folin–Ciocalteu colorimetric method according to Singleton and Rossi, with slight modifications [29]. Cocoa bean samples were ground into a fine powder, and approximately 1.0 g of each sample was accurately weighed into a 125 mL Erlenmeyer flask. Ten milliliters of 80% (*v*/*v*) methanol were added, and the mixture was extracted by shaking at room temperature for 60 min. The extract was filtered through Whatman No. 4 filter paper, and the filtrate was collected for analysis.

An aliquot (60 μL) of the extract was transferred into a test tube in triplicate, followed by the addition of 2.5 mL of Folin–Ciocalteu reagent. The mixture was vortex-mixed and allowed to react in the dark for 2 min. Subsequently, 2.0 mL of sodium carbonate solution was added, and the mixture was thoroughly mixed before incubation at 50 °C for 15 min. After incubation, the reaction mixture was immediately cooled to room temperature, and the absorbance was measured at 760 nm using a UV–Vis spectrophotometer. Eighty percent methanol was used as the blank.

A calibration curve was prepared using gallic acid as the standard, and the results were expressed as milligrams of gallic acid equivalents per gram of sample (mg GAE/g sample).

$$\mathrm{TPC}\;(\mathrm{mg}\;\mathrm{GAE}/g)=\frac{C\times V}{M}$$

where *C* is concentration of gallic acid (mg/mL) from the calibration curve, *V* is volume of extract (mL) used in the reaction and *M* is mass of the sample (g) extracted.

#### 2.2.5. Determination of Total Titratable Acidity

Total titratable acidity (TA) was determined according to the AOAC International Official Method 942.15 [27]. Briefly, 5 g of ground cocoa sample was homogenized with distilled water, and the volume was adjusted to 100 mL in a volumetric flask. The extract was filtered through Whatman No. 1 filter paper. An aliquot of 10 mL of the filtrate was transferred into a 125 mL Erlenmeyer flask and mixed with approximately 50 mL of distilled water. Two to three drops of 1% phenolphthalein indicator were added, and the solution was titrated with standardized 0.1 N sodium hydroxide (NaOH) to a persistent pale pink endpoint (approximately 30 s). Total titratable acidity was calculated and expressed as percentage of acetic acid using the following equation:

$$\mathrm{TA}\;\left(\%\;\mathrm{as}\;\mathrm{acetic}\;\mathrm{acid}\right)=\frac{V\;\times \;N\;\times \;0.060\;\times \;D\;\times \;100}{W\;\times \;1000}$$

where *V* is the volume of NaOH used (mL), *N* is the normality of NaOH, 0.060 is the equivalent weight of acetic acid, *D* is the dilution factor, and *W* is the sample weight (g).

#### 2.2.6. Determination of Total Sugar, Lactic Acid, Acetic Acid and Ethanol Content

Approximately 25 g of ground fermented cocoa beans were extracted with 100 mL of distilled water, filtered through two layers of cheesecloth, and subsequently passed through a 0.20 μm nylon membrane filter prior to HPLC analysis. Identification of glucose, fructose, sucrose (total sugar), lactic acid, acetic acid, and ethanol was based on comparison of retention times with authentic standards. Quantification was carried out using external calibration curves prepared from analytical-grade standards. The results were expressed as mg/g (wet basis).

Glucose, fructose, sucrose, lactic acid, acetic acid, and ethanol were determined using a high-performance liquid chromatography (HPLC) system (Chromaster, Hitachi High-Tech, Tokyo, Japan) equipped with a refractive index detector (RI Detector 5450, Hitachi, Ibaraki, Japan). Separation was performed on an Aminex^®^ HPX-87H column (300 × 7.8 mm i.d.; Bio-Rad Laboratories, Hercules, CA, USA) [30]. The mobile phase consisted of 5 mM sulfuric acid (H_2_SO_4_) prepared with HPLC-grade water and was delivered at a flow rate of 0.75 mL/min. The column temperature was maintained at 40 °C, and the injection volume was 10 µL. The total run time was 25 min per sample.

#### 2.2.7. Determination of Theobromine and Catechin Content

The theobromine and catechin contents were determined using high-performance liquid chromatography (HPLC) according to a modified method of Srdjenovic et al. [31]. Five grams of cocoa bean sample were extracted with 50 mL of a 50:50 (*v*/*v*) deionized water–ethanol solution by shaking for 10 min. The extract was filtered through Whatman No. 4 filter paper followed by a 0.20 μm nylon membrane filter prior to HPLC analysis. Separation was performed on an Agilent ZORBAX C18 column (4.6 × 250 mm, 3.5 μm) maintained at 30 °C. The mobile phase consisted of 0.1% (*v*/*v*) trifluoroacetic acid in water (solvent A) and HPLC-grade methanol (solvent B) using gradient elution at a flow rate of 1.0 mL/min. The injection volume was 5 μL, and the total run time was 25 min. Detection was performed using a diode-array detector at 210 nm. Theobromine and catechin were identified by comparison with authentic standards based on their retention times, and quantification was performed using external calibration curves. The results were expressed as mg/g (dry basis).

#### 2.2.8. Statistical Analyses

All experiments were conducted using a completely randomized design (CRD) with four fermentation temperature treatments (15, 25, 35 and 45 °C). Each treatment was performed in triplicate, and all results are presented as the mean ± standard deviation (SD). During cocoa bean fermentation, temperature, pH, moisture content, total soluble solids, titratable acidity, reducing sugar, total phenolic content, Hunter L*a*b* color values (around the seed), total sugar, lactic acid, acetic acid, and ethanol were determined every two days for a total fermentation period of 8 days. The changes in these parameters during fermentation were presented as kinetic profiles. Following fermentation and drying, dried cocoa beans were analyzed for yield, total soluble solids, Hunter L*a*b* color values, water activity (a_w_), moisture content, total acid, reducing sugar, total phenolic content, theobromine, and catechin. Data obtained from dried cocoa beans were analyzed by one-way analysis of variance (one-way ANOVA) to determine the effect of fermentation temperature. Mean comparisons were performed using Tukey’s multiple comparison test, and differences were considered statistically significant at *p* < 0.05. All data processing and statistical evaluations were conducted using Minitab statistical software (Version 21, Minitab Inc., State College, PA, USA).

## 3. Results and Discussion

### 3.1. Kinetic Characterization of Cocoa Bean Fermentation at Different Temperatures (15, 25, 35, and 45 °C)

#### 3.1.1. Changes in Physicochemical Properties During Fermentation

During cocoa fermentation at different controlled temperatures (15, 25, 35, and 45 °C), with daily turning of the cocoa beans, the moisture content of beans fermented at 25, 35, and 45 °C gradually decreased throughout the fermentation period (Figure 1 and Figure S1). By day 8, the moisture content for these treatments ranged from 20.72% to 28.66%, whereas the beans fermented at 15 °C retained a significantly higher moisture content of 49.81%. This trend can be attributed to the increasing and subsequently stable temperatures maintained within the fermentation containers (Figure 2 and Figure S2), which enhanced moisture evaporation and promoted the degradation and liquefaction of the surrounding pulp, thereby accelerating moisture loss from the beans [32,33,34]. In contrast, the temperature of the cocoa beans fermented at 15 °C remained consistently low throughout the fermentation process. This suggests that such a low temperature restricted the rate of pulp degradation and moisture evaporation, leading to less extensive physicochemical changes compared to the higher-temperature treatments [25]. Under conventional conditions, fermentation temperatures typically range between 25 and 30 °C, which naturally supports optimal pulp degradation and the associated chemical transformations [6,9].

The reducing sugar content of cocoa beans fermented at different controlled temperatures decreased progressively with increasing fermentation time. Among the treatments, cocoa bean mass fermented at 45 °C exhibited the most pronounced reduction in reducing sugar content, reaching 5.13 mg/g on day 8. This was followed by the bean mass fermented at 35, 25, and 15 °C, with final reducing sugar contents of 8.77, 11.15, and 15.89 mg/g, respectively (Figure 3 and Figure S3). This progressive decline is primarily driven by inherent biochemical processes and microbial activities typically associated with cocoa fermentation, where sugars are utilized as primary carbon sources for the production of ethanol and organic acids [9,35,36]. These results were consistent with the changes observed in total soluble solids (TSS) (Figure 4 and Figure S4), which also showed a continuous decline throughout the fermentation period. The lowest TSS value was observed in cocoa beans fermented at 45 °C, decreasing to 3.15 °Brix on day 8. The accelerated reduction in both reducing sugars and TSS at higher temperatures (35–45 °C) further indicates an enhanced enzymatic and microbial reaction rate, which facilitates efficient pulp degradation and optimal chocolate flavor precursor development compared to lower-temperature treatments [18,25].

The pH values of wet cocoa beans fermented at controlled temperatures of 15, 25, 35, and 45 °C remained relatively stable throughout the 8-day fermentation period. The initial pH of fresh cocoa beans was 5.3, while the final pH values on day 8 ranged from 4.7 to 5.2 (Figure 5 and Figure S5). In contrast, total titratable acidity varied among the temperature treatments. On day 8, wet cocoa beans fermented at 45 °C exhibited the highest total titratable acidity (0.35%) (Figure 6 and Figure S6). This trend may be associated with fermentation conditions at 45 °C, which is within the temperature range reported to favor acetic acid bacteria (AAB) [37]. Previous studies have suggested that AAB can facilitate the oxidation of ethanol to acetic acid, potentially contributing to acid accumulation during cocoa fermentation [6,18]. However, because microbial populations and individual organic acids were not determined in the present study, the contribution of AAB could not be confirmed. In contrast, beans fermented at 15 °C showed the lowest total titratable acidity (0.20%) on day 8 (Figure 6). This finding may reflect slower fermentation-related processes and lower acid production at lower temperatures, as reported previously [14,38].

The total phenolic content (TPC) of wet cocoa beans fermented at different controlled temperatures decreased progressively as fermentation time increased. On day 8, the beans fermented at 45 °C exhibited the lowest TPC, with a value of 0.13 mg/g, whereas beans fermented at 15 °C retained the highest TPC among all treatments (Figure 7 and Figure S7). These findings indicate that higher fermentation temperatures promote the degradation of phenolic compounds in cocoa beans. The accelerated reduction in TPC at elevated temperatures is typically associated with the heat-induced diffusion of polyphenols out of the cellular vacuoles, combined with enhanced enzymatic oxidation by polyphenol oxidase and subsequent non-enzymatic polymerization into complex tannins [20,39]. Similar observations regarding this downward trend have been reported by Brito et al. [40], who found that the total phenolic content decreased with increasing fermentation time. After 7 days of fermentation, the total phenolic content was reduced by approximately 31%, while anthocyanins, one of the major phenolic compounds responsible for the purple color of cocoa beans, decreased by as much as 79% [41].

The color evolution of cocoa beans was monitored both at the surface and within the cotyledons (cross-section) throughout the 8-day fermentation process (Figure 8 and Figure 9). Regarding the surface color, the lightness (L* value) gradually decreased across all temperature treatments, reflecting the condensation of monomeric polyphenols into dark-colored complex molecules [20,42]. While temperature had a minor effect on L* surface values, it significantly influenced redness (a* value). Beans fermented at 45 °C exhibited the highest a* value (8.03), attributed to heat-induced enzymatic oxidation forming reddish-brown phlobaphenes [11,43]. Yellowness (b* value) remained relatively stable, consistent with well-aerated fermentation patterns [12,44] (as further detailed in Figure S8).

In contrast, the cotyledon color (cross-section) showed more pronounced variations. As shown in Figure 9 and Figure S9, the L* values of the cotyledons decreased more markedly than those at the surface, particularly at 35 and 45 °C, indicating more pronounced color changes within the bean tissue. The a* and b* values of the cotyledons generally increased during fermentation, and the magnitude of these changes differed among the temperature treatments. Additionally, specific dynamics of internal cotyledon color parameters, pH, and total titratable acidity between days 6 and 8 are summarized in Table S1. These results suggest that fermentation temperature influenced color development in both the bean surface and cotyledons. However, further analyses are required to clarify the mechanisms responsible for these color changes.

A limitation of the current study is the absence of a standard ‘cut test’ to evaluate the final internal color development (i.e., degree of brown/purple cotyledon differentiation) of the fermented cocoa beans. Consequently, while the kinetic profiles presented herein effectively characterize the fermentation process, they do not directly correlate these physicochemical changes with the final physical quality of the cotyledons. Future research should integrate cut-test evaluations and sensory analysis to comprehensively elucidate the relationship between fermentation kinetics and final product quality.

#### 3.1.2. Changes in Fermentation Metabolites During Fermentation

The kinetic changes in sugar consumption and metabolite production during cocoa bean fermentation at 15, 25, 35, and 45 °C are expressed in Figure 10, Figure 11, Figure 12 and Figure 13 (specifically supporting Figures S10–S13). Fermentation at 15, 25, and 35 °C showed rapid sugar consumption during the first two days of fermentation, whereas sugar utilization at 45 °C was comparatively slower but continued steadily from day 0 to day 6 (Figure 10 and Figure S10). This pattern may be related to differences in fermentation activity among the temperature treatments. Previous studies have reported that yeasts play an important role in sugar utilization during the initial stage of cocoa fermentation and generally grow optimally at 25–30 °C [1,2]. Therefore, the slower decrease in sugar content at 45 °C may reflect less favorable conditions for sugar utilization during the early fermentation stage [6,45]. Consistent with this trend, ethanol production during the first two days of fermentation was lower at 35 and 45 °C than at 15 and 25 °C (Figure 11 and Figure S11). These results suggest that temperature influenced the early fermentation process and metabolite production [46]. However, as microbial populations were not determined in this study, the contribution of yeasts to these changes cannot be confirmed.

Ethanol production reached its maximum on day 2, particularly in the 15 and 25 °C treatments (Figure 11 and Figure S11). This pattern may be associated with conditions that favor ethanol production during the early anaerobic stage of fermentation, as reported in previous studies [25,34]. As fermentation progressed, ethanol concentrations declined while acetic acid concentrations increased. This trend is consistent with the conversion of ethanol to acetic acid during cocoa fermentation under increasingly aerobic conditions [6,16,46]. Previous studies have described a transition from alcoholic fermentation during the early stage to acetic acid production during the later stage as a characteristic feature of cocoa fermentation. Such changes have been reported to be associated with bean death, enzymatic activation, and the subsequent development of cocoa flavor precursors [1,47].

Lactic acid production increased during the middle stage of fermentation, with the highest accumulation occurring from 2 to 4 days at 25 °C, and from 4 to 6 days at 15 and 35 °C (Figure 12 and Figure S12). This increase may be associated with changes in fermentation activity during the intermediate stage. Previous studies have reported that LAB can contribute to lactic acid production through the conversion of sugars during cocoa fermentation [18,48]. The differences in the timing of lactic acid accumulation among the temperature treatments may indicate that temperature influenced the rate of fermentation-related processes. These findings are consistent with those reported by Wood and Lass [49], who described an increase in LAB during the intermediate stage of cocoa fermentation. Lactic acid production has been reported to contribute to changes in acidity and internal bean properties during fermentation [6,46].

Acetic acid concentrations increased markedly between days 2 and 4, particularly at 25 and 35 °C, before declining toward the end of fermentation (Figure 13 and Figure S13). This trend may be associated with the conversion of ethanol to acetic acid under increasingly aerobic conditions, as reported during cocoa fermentation [9,25]. In the present study, the fermentation mass was turned daily after the initial 48 h, which may have increased oxygen availability and contributed to the observed increase in acetic acid during days 2–4. The increase in acetic acid was also temporally associated with ethanol production during the preceding stage of fermentation. This conversion has been reported to be an exothermic process and may contribute to heat generation within the fermenting mass [16].

Acetic acid accumulation has been reported to contribute to acidification and biochemical changes within cocoa bean cotyledons, which may be associated with cellular disruption, enzyme activity, and the subsequent formation of flavor precursors during fermentation and roasting [18,50]. Nevertheless, further analyses of microbial populations, organic acids, and bean biochemical changes are required to clarify these mechanisms under the present fermentation conditions [2,36].

### 3.2. Physicochemical Properties of Dried Cocoa Beans After Fermentation at Different Temperatures

The effects of fermentation temperatures (15, 25, 35, and 45 °C) on the physical and chemical qualities of dried cocoa beans were investigated. The results showed that fermentation temperature significantly affected yield, total soluble solids (TSS), lightness (L* value), water activity (*a*_w_), moisture content, total titratable acidity, reducing sugar, and total phenolic content, as presented in Table 1 and Table 2. Fermentation at 15 °C resulted in the highest yield (47.17%), followed by fermentation at 25 and 35 °C, whereas the lowest yield (37.67%) was obtained at 45 °C. This progressive decrease in yield at higher temperatures can be attributed to the accelerated degradation and liquefaction of the surrounding mucilaginous pulp, leading to a greater loss of pulp by-products during the turning and drainage of sweating compared to lower-temperature treatments [34,51]. Regarding total soluble solids, fermentation at 15, 25, and 35 °C produced the highest TSS values, with no significant differences among these treatments (*p* > 0.05), recording 3.27, 3.07, and 2.80 °Brix, respectively. For color characteristics, fermentation at 15 °C produced dried cocoa beans with the highest lightness (L* value) of 47.44. In contrast, the redness (a* value) and yellowness (b* value) of the dried beans were not significantly affected by fermentation temperature (*p > 0.05*), with a* values ranging from 14.57 to 19.44 and b* values ranging from 15.00 to 22.16. This relative stability in chromatic values (a* and b*) among the dried end-products aligns with typical physical transformations observed during standard convective drying, where final surface coloration is strongly governed by moisture removal constraints [12,20]. Regarding water activity (a_w_), the highest value (0.57) was observed in cocoa beans fermented at 15 °C, as shown in Table 1. This significantly higher a_w_ retention at 15 °C indicates a restricted evaporation rate during processing, which is closely linked to the incomplete breakdown of the hydrophilic pulp matrix under cold fermentation constraints [18,52].

Analysis of moisture content showed that fermentation at 15 and 25 °C resulted in the highest moisture contents of dried cocoa beans, with values of 6.26% and 5.51%, respectively. In contrast, dried cocoa beans fermented at 25, 35, and 45 °C showed significantly higher total titratable acidity than those fermented at 15 °C, with no significant differences among the three higher-temperature treatments (Table 2). Cocoa fermentation is generally initiated at ambient temperatures of approximately 25–30 °C, during which metabolic processes associated with fermentation may cause the fermentation temperature to increase to 35–40 °C within the first 48 h [6]. These temperature conditions have been reported to be associated with organic acid production and subsequent pulp degradation during cocoa fermentation [18,34].

The reducing sugar content was highest in cocoa beans fermented at 15 °C, reaching 17.84 mg/g. Similarly, the total phenolic content in this treatment was also the highest among all treatments, with a value of 2.39 mg/g (Table 2). This pattern may be associated with the relatively low fermentation temperature, which may have resulted in less extensive fermentation-related changes. Consequently, the lower reduction in reducing sugars and phenolic compounds naturally present in fresh cocoa beans may have contributed to the higher concentrations of these compounds in the final dried beans [53].

The concentrations of theobromine and catechin in dried cocoa beans fermented at different temperatures were also determined. No significant differences (*p* > 0.05) were observed in theobromine content among treatments, with values ranging from 3.38 to 3.87 mg/g. This relatively stable theobromine content observed after fermentation and drying may be attributed to the inherent stability of its purine alkaloid structure under the moderate temperatures typically employed during the fermentation and convective drying processes [1,54]. Catechin content ranged from 0.03 to 0.10 mg/g, with the highest value observed in cocoa beans fermented at 45 °C; however, this value was not significantly different (*p* > 0.05) from those obtained at 25 and 35 °C (Table 2). Previous studies have reported that catechins may undergo oxidation and other transformations during cocoa fermentation and processing [20,55]. Therefore, the catechin contents observed among the temperature treatments may reflect differences in the extent of these changes during fermentation and drying.

## 4. Conclusions

The present study demonstrated that controlled fermentation temperature influenced both the fermentation kinetics and the final quality of dried cocoa beans. During the 8-day fermentation period, lower-temperature fermentation at 15 °C was associated with less pronounced changes in reducing sugars, total soluble solids, and organic acid profiles. In contrast, fermentation at higher temperatures, particularly 35 and 45 °C, showed greater changes in these fermentation-related parameters, including a greater reduction in reducing sugars and an increase in total titratable acidity. The observed ethanol, lactic acid, and acetic acid profiles further indicated that temperature conditions and daily turning after the first 48 h affected the progression of fermentation-related metabolic changes. After fermentation and subsequent hot-air drying, the final dried cocoa beans produced at 15 °C retained higher moisture, reducing sugar, and total phenolic compound contents. In comparison, the dried beans produced under higher fermentation temperatures showed lower reducing sugar and total phenolic compound contents, as well as higher total titratable acidity. Theobromine content remained stable among the dried-bean treatments, whereas catechin content varied with fermentation temperature. Temperature conditions also influenced the surface color and water activity of the final dried cocoa beans.

Overall, controlled temperature fermentation may be applied as a practical postharvest management approach to regulate fermentation progression and improve the consistency of final dried cocoa bean quality. Lower temperatures may be useful when retaining phenolic compounds and reducing sugars is desirable, whereas higher temperatures may promote more extensive fermentation-related changes and acidity development. However, because microbial populations and enzymatic activities were not determined, the involvement of specific microorganisms in the observed metabolite changes could not be confirmed. Further studies integrating microbial analysis, cut-test, enzymatic activity, sensory evaluation, and larger-scale trials are needed to establish the most suitable temperature conditions for smallholder cocoa-processing systems.

## Figures and Tables

**Figure 1:**
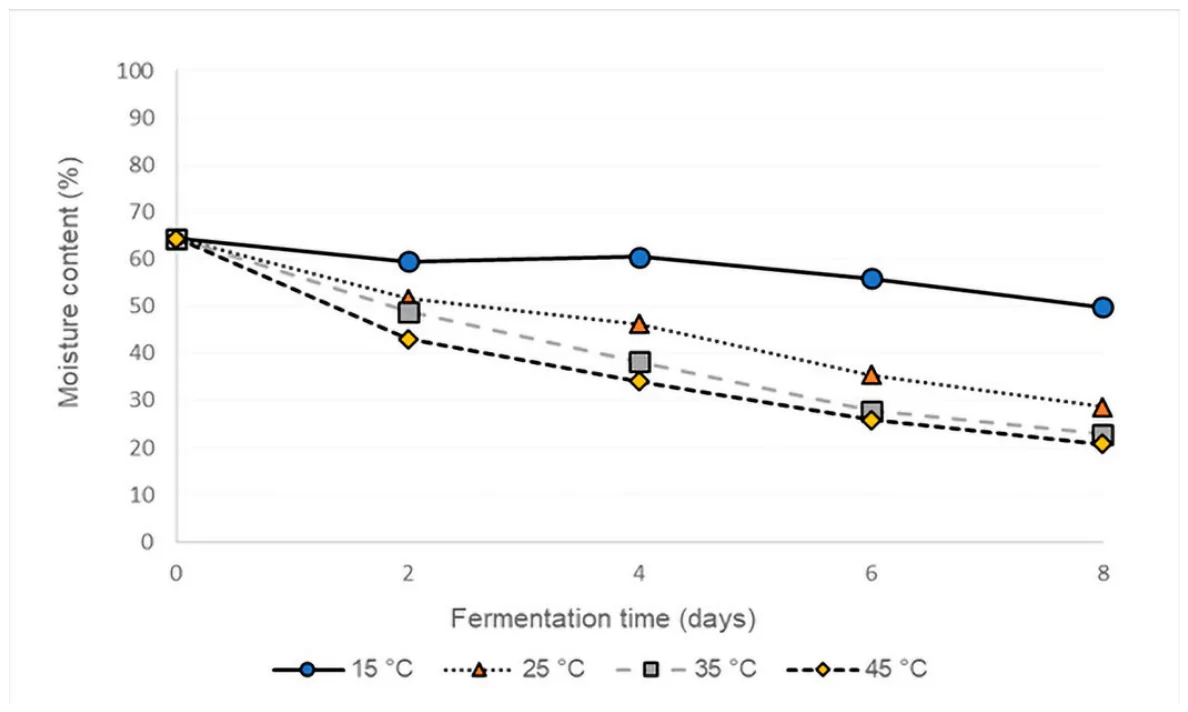
Changes in the moisture content of cocoa beans during fermentation at different fermentation temperatures (15, 25, 35, and 45 °C). Data are expressed as mean ± SD (*n* = 3).

**Figure 2:**
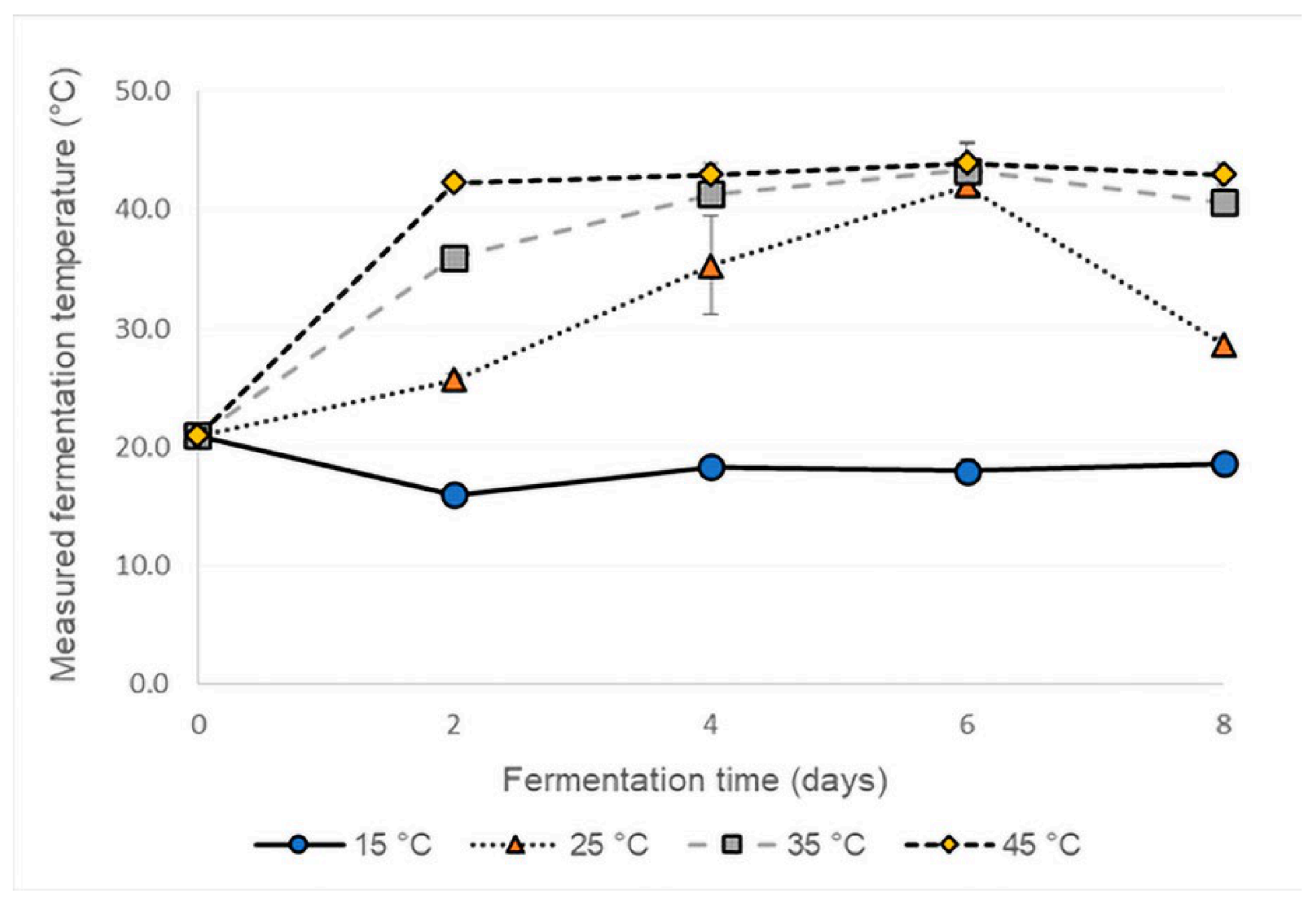
Changes in the measured fermentation temperature of cocoa beans during fermentation at different fermentation temperatures (15, 25, 35, and 45 °C). Data are expressed as mean ± SD (*n* = 3).

**Figure 3:**
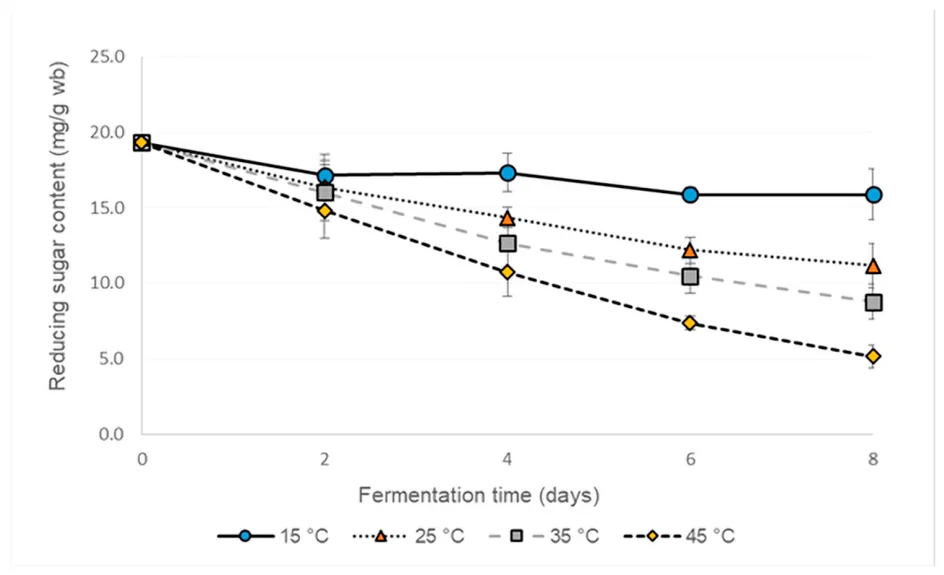
Changes in the reducing sugar content of cocoa beans during fermentation at different fermentation temperatures (15, 25, 35, and 45 °C). Data are expressed as mean ± SD (*n* = 3). wb: wet basis.

**Figure 4:**
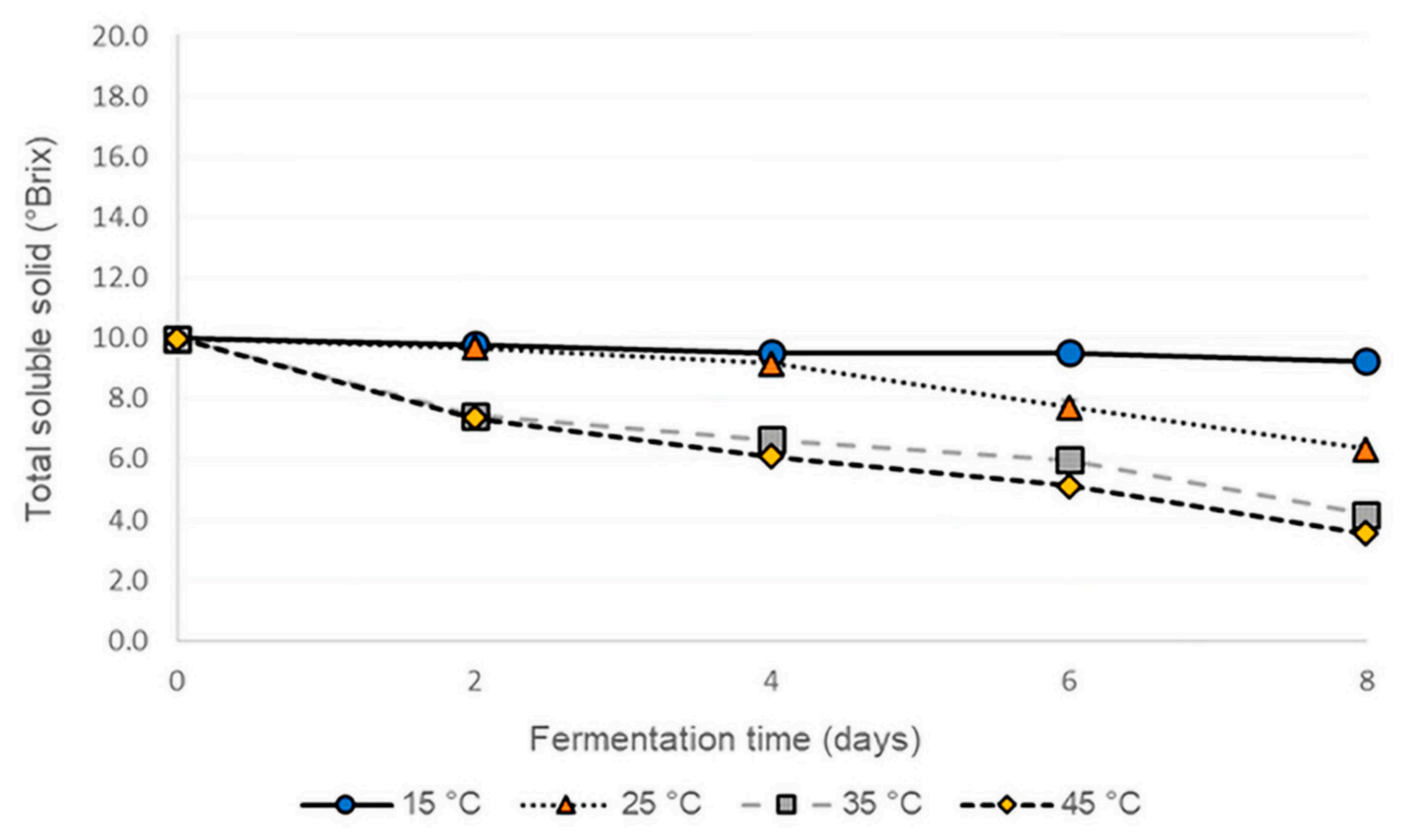
Changes in the total soluble solid content of cocoa beans during fermentation at different fermentation temperatures (15, 25, 35, and 45 °C). Data are expressed as mean ± SD (*n* = 3).

**Figure 5:**
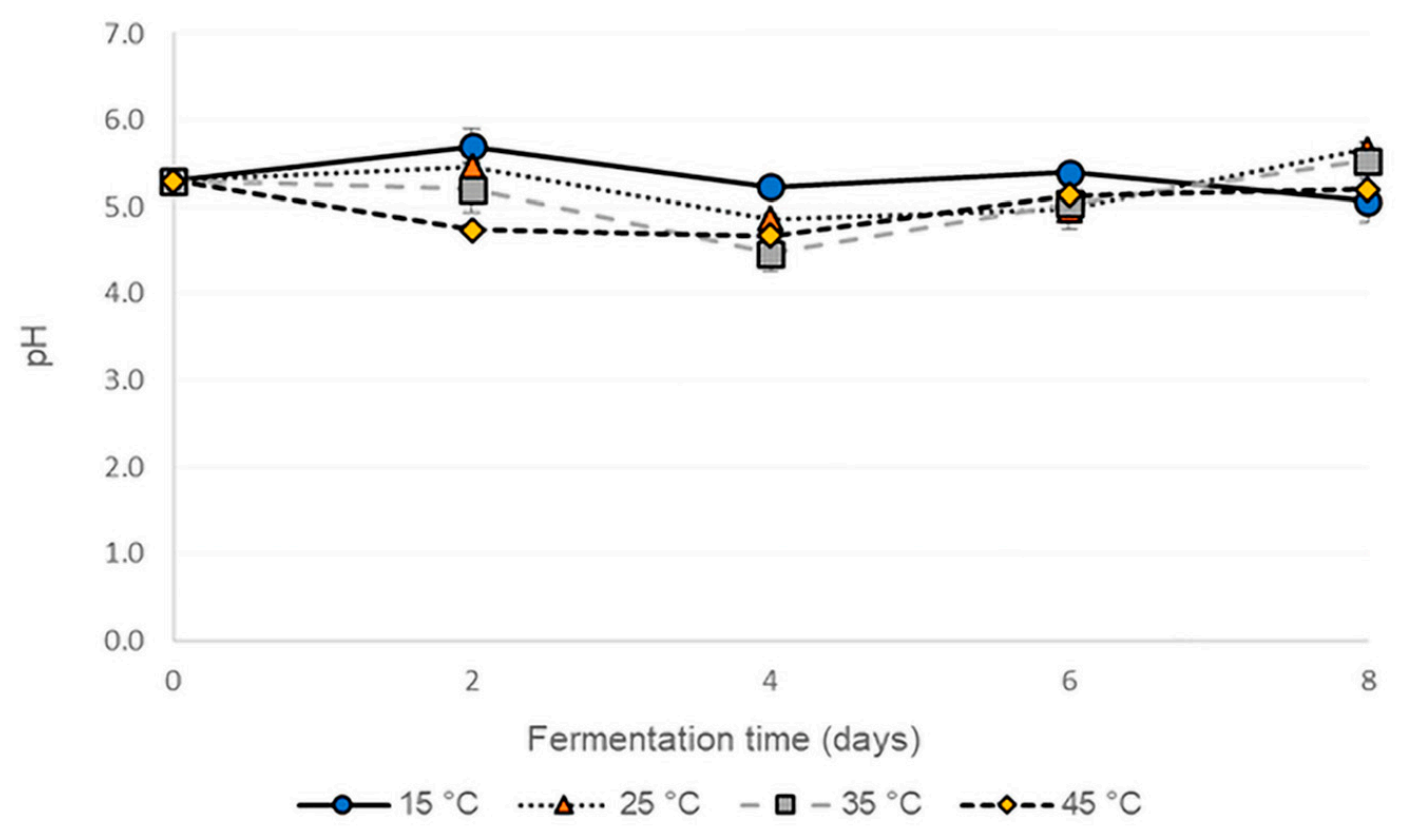
Changes in the pH of cocoa beans during fermentation at different fermentation temperatures (15, 25, 35, and 45 °C). Data are expressed as mean ± SD (*n* = 3).

**Figure 6:**
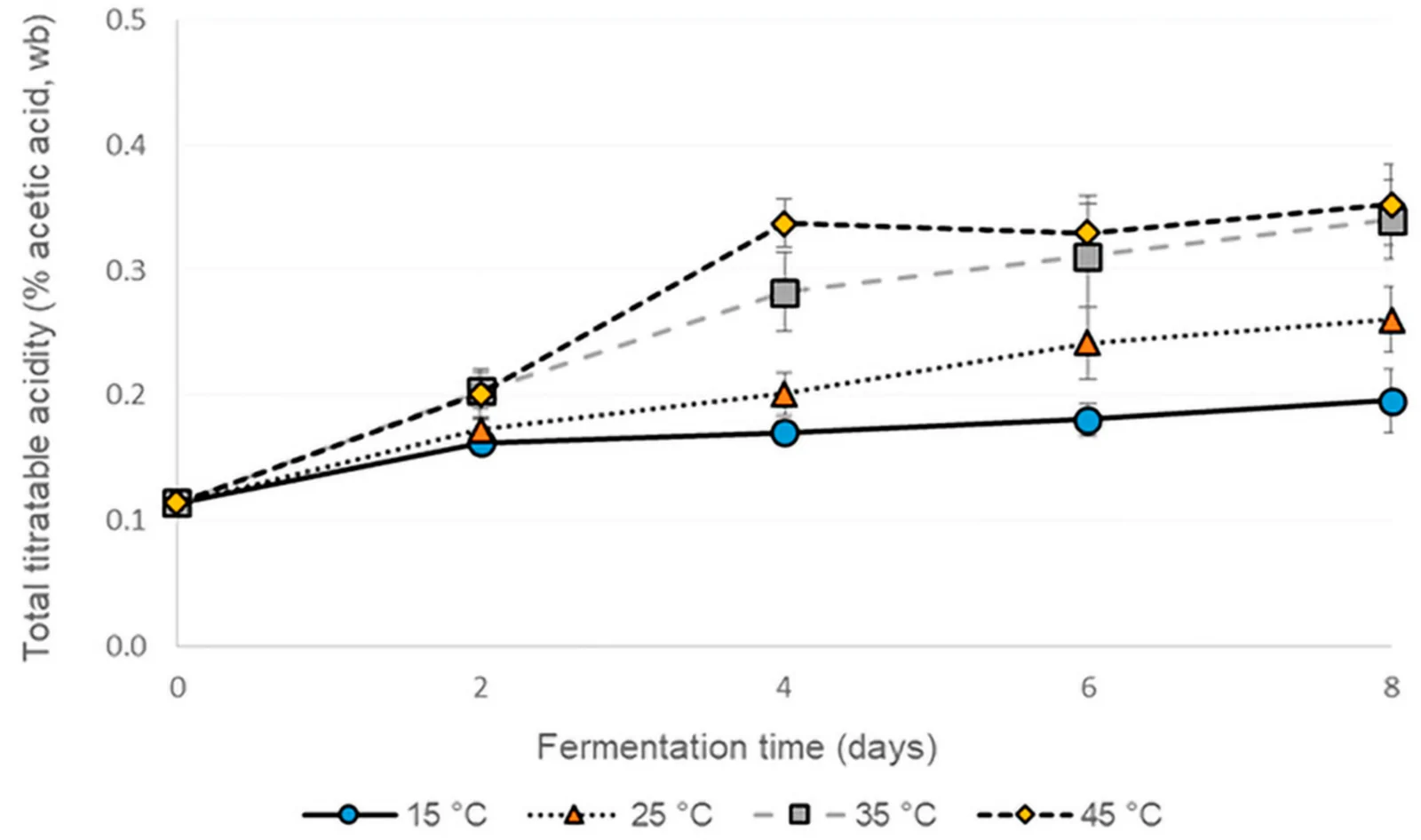
Changes in the total titratable acidity (%acetic acid) of cocoa beans during fermentation at different fermentation temperatures (15, 25, 35, and 45 °C). Data are expressed as mean ± SD (*n* = 3). wb: wet basis.

**Figure 7:**
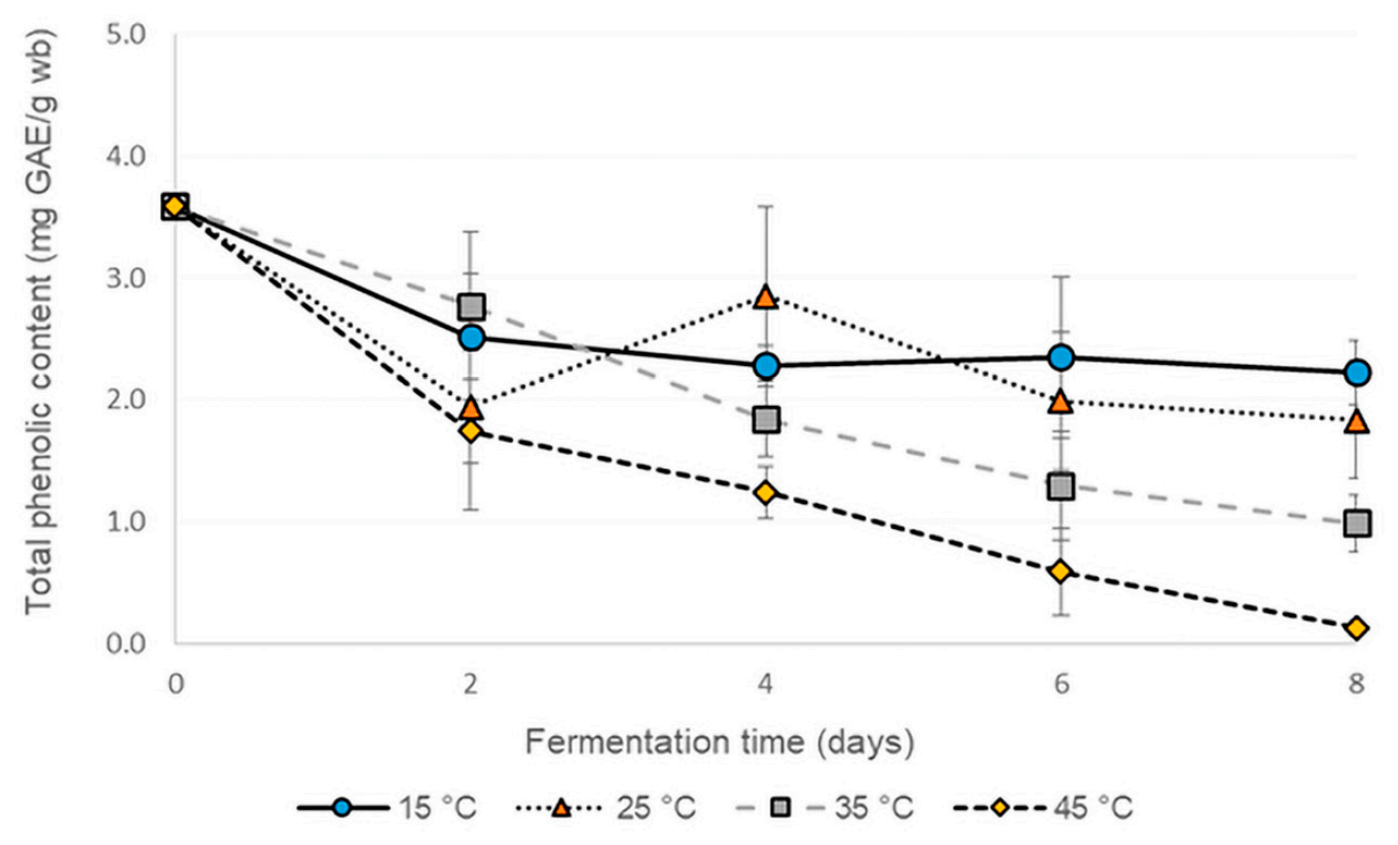
Changes in the total phenolic content of cocoa beans during fermentation at different fermentation temperatures (15, 25, 35, and 45 °C). Data are presented as mean ± SD (*n* = 3). wb: wet basis.

**Figure 8:**
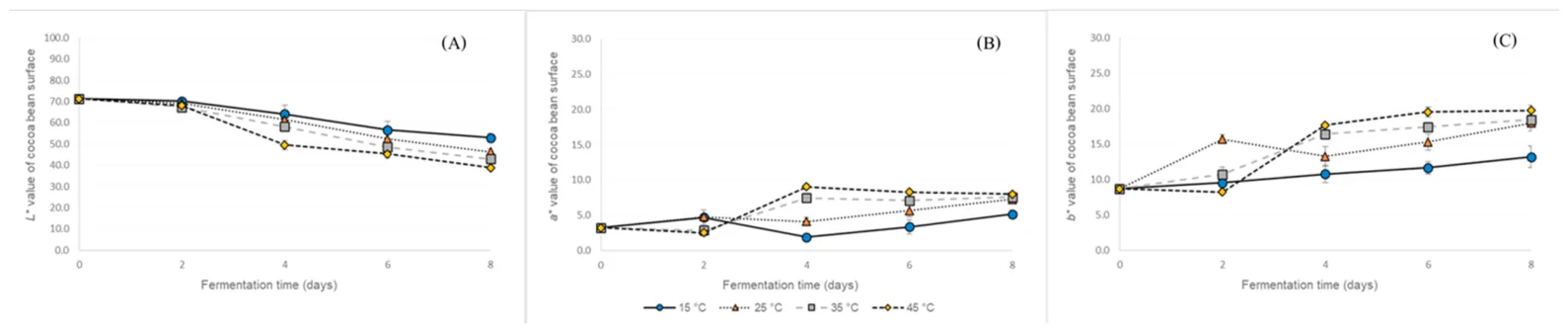
Changes in the L*a*b* color parameters of cocoa bean surface during fermentation at different fermentation temperatures (15, 25, 35, and 45 °C). (**A**) L* value, (**B**) a* value, and (**C**) b* value. Data are presented as mean ± SD (*n* = 3).

**Figure 9:**
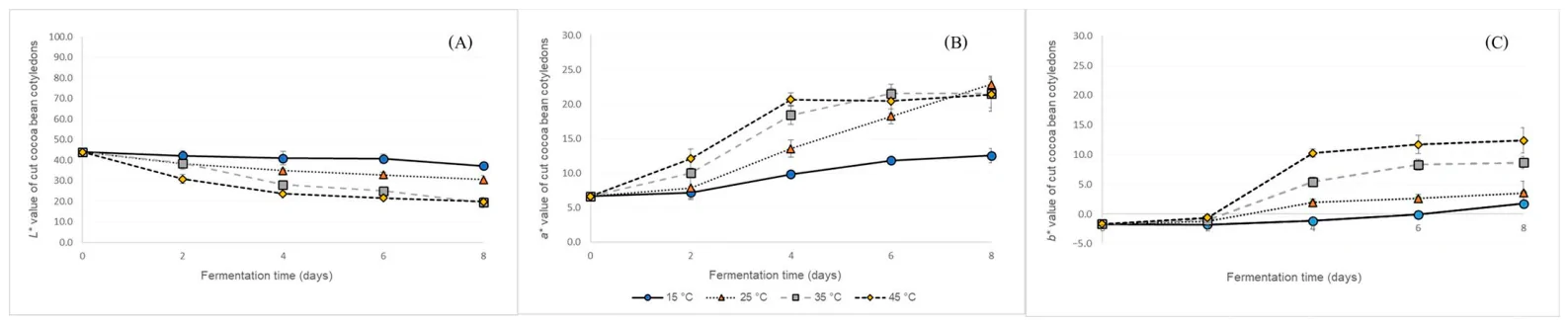
Changes in the L*a*b* color parameters of cocoa bean cotyledons during fermentation at different fermentation temperatures (15, 25, 35, and 45 °C). (**A**) L* value, (**B**) a* value, and (**C**) b* value. Data are expressed as mean ± SD (*n* = 3).

**Figure 10:**
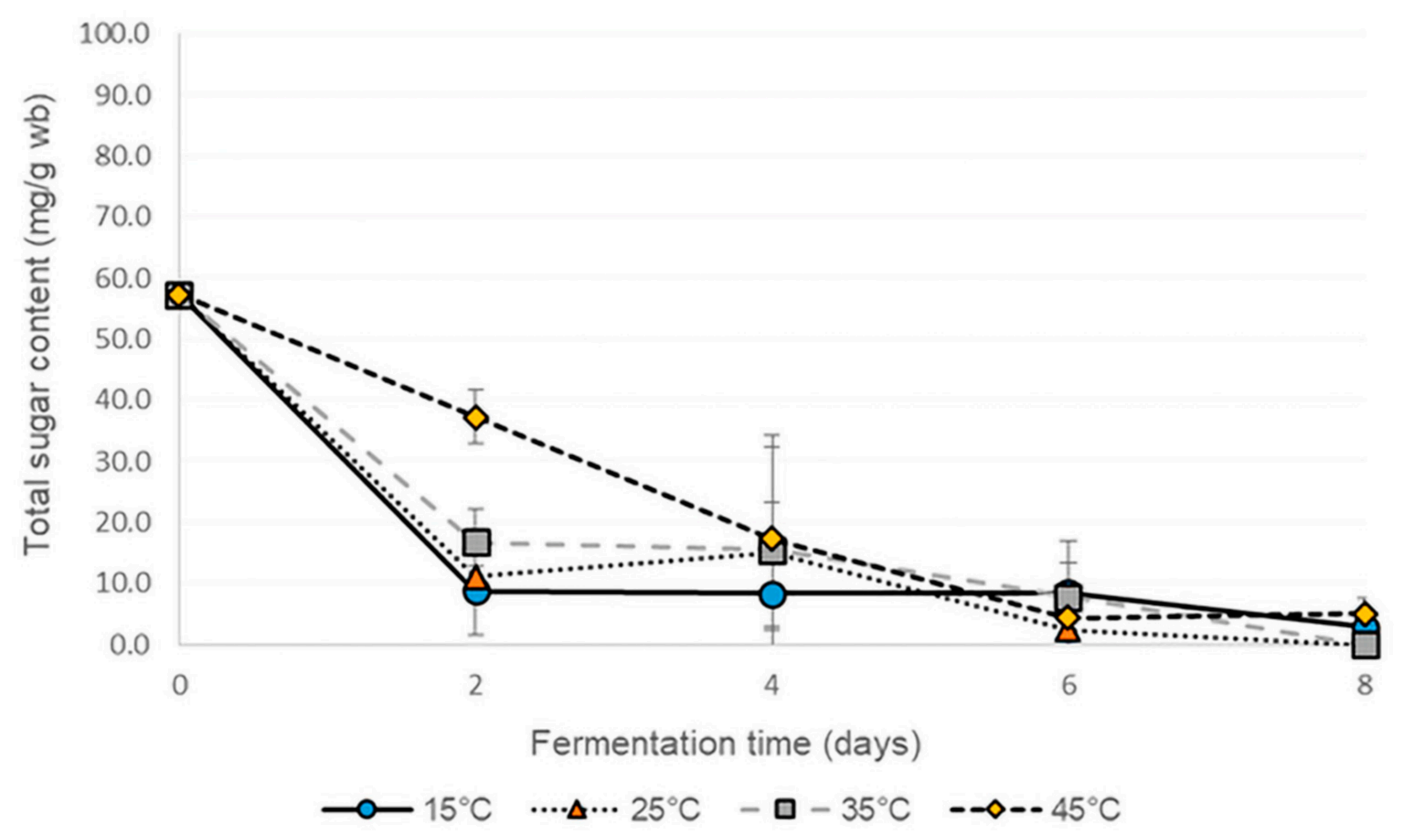
Changes in total sugar content of cocoa beans during fermentation at different fermentation temperatures (15, 25, 35, and 45 °C). Data are expressed as mean ± SD (*n* = 3). wb: wet basis.

**Figure 11:**
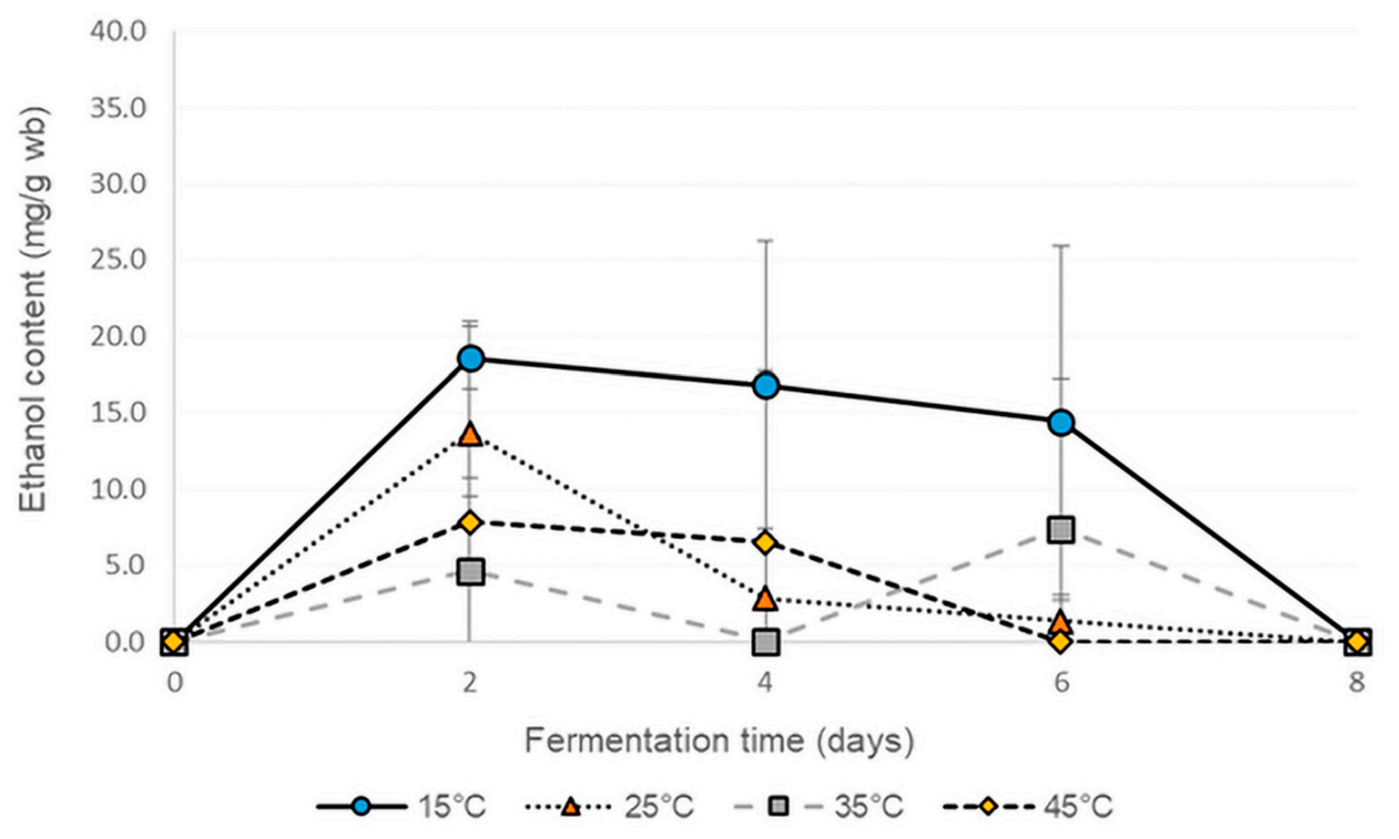
Changes in ethanol content of cocoa beans during fermentation at different fermentation temperatures (15, 25, 35, and 45 °C). Data are expressed as mean ± SD (*n* = 3). wb: wet basis.

**Figure 12:**
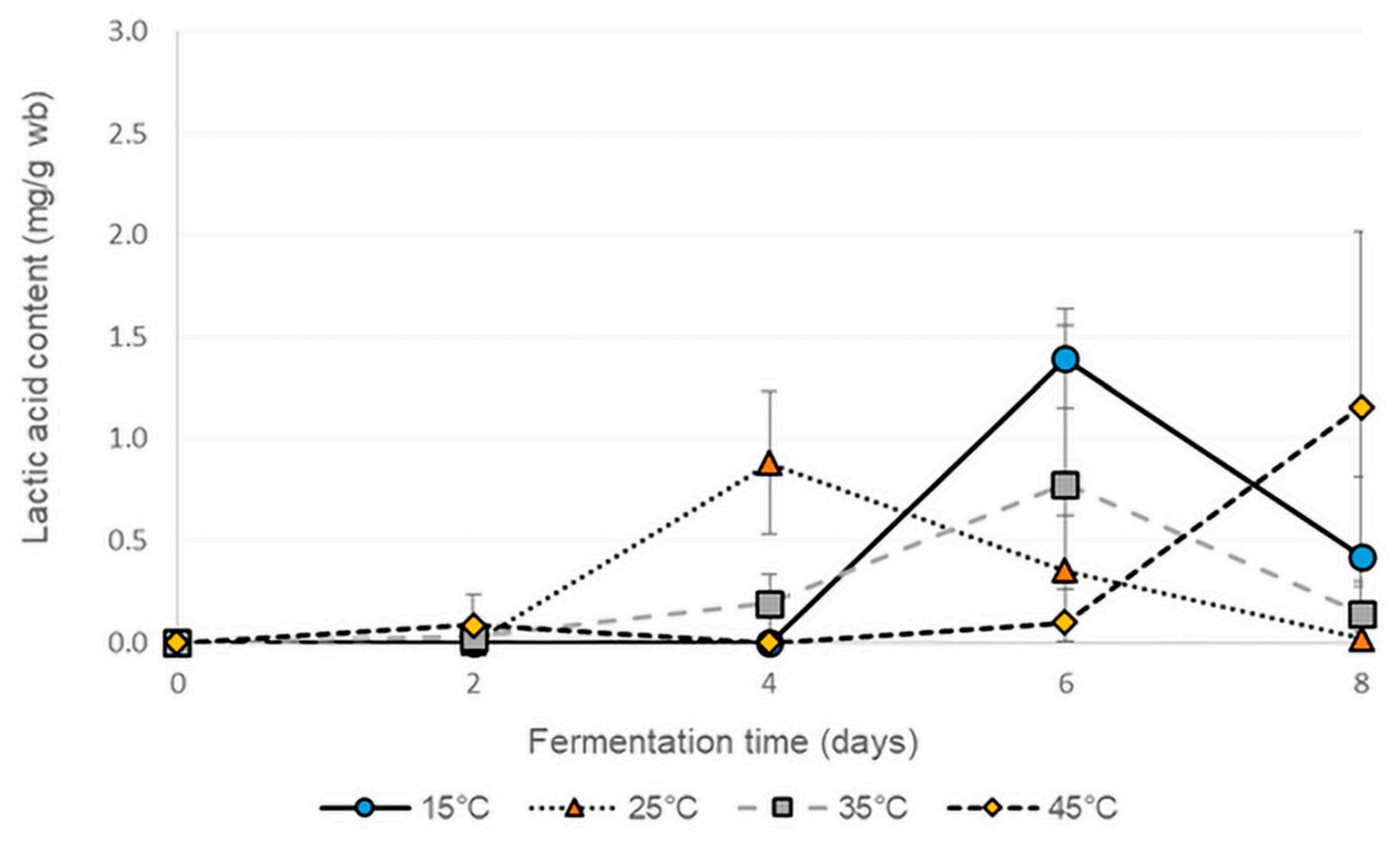
Changes in lactic acid content of cocoa beans during fermentation at different fermentation temperatures (15, 25, 35, and 45 °C). Data are expressed as mean ± SD (*n* = 3). wb: wet basis.

**Figure 13:**
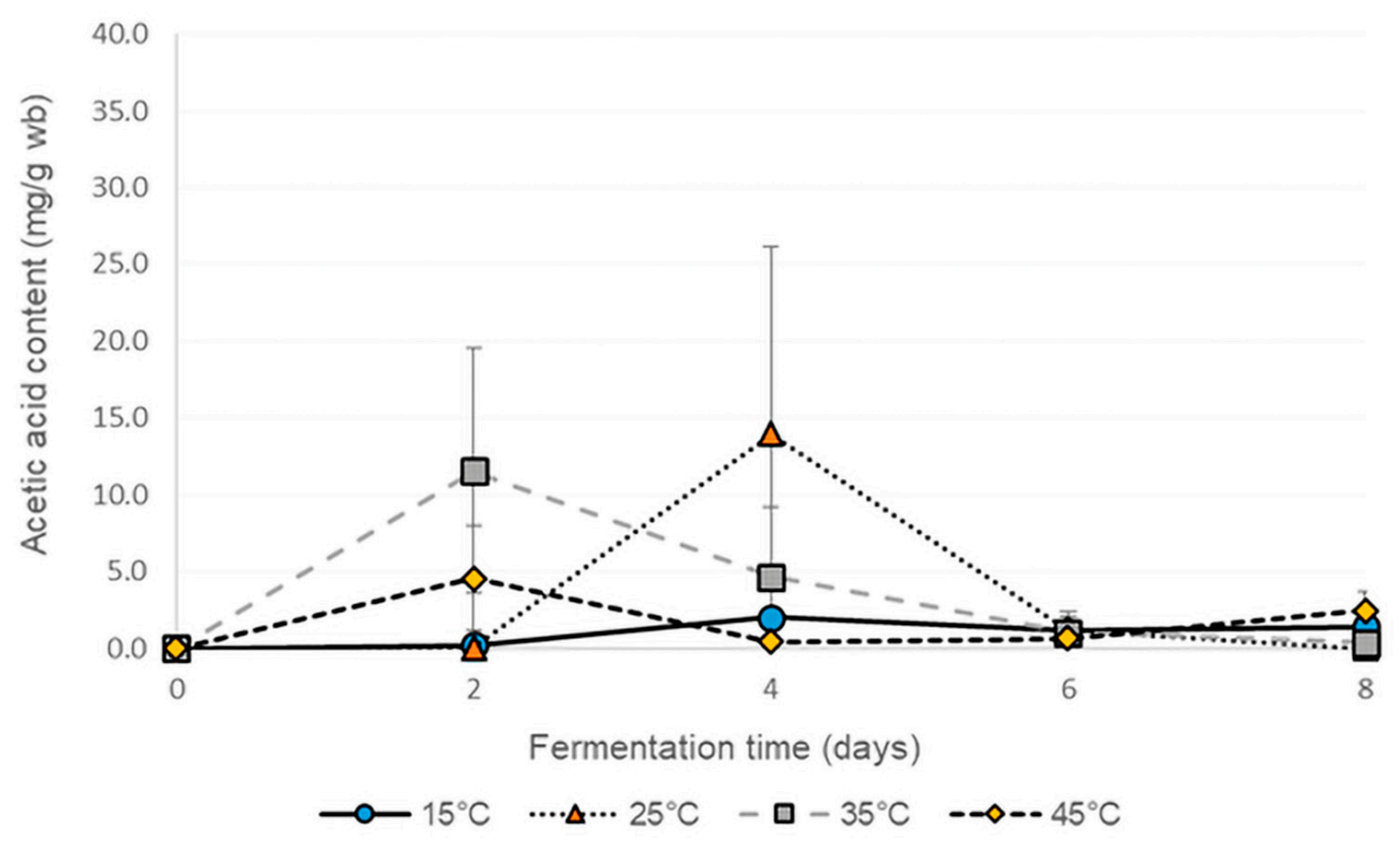
Changes in acetic acid content of cocoa beans during fermentation at different fermentation temperatures (15, 25, 35, and 45 °C). Data are expressed as mean ± SD (*n* = 3). wb: wet basis.

**Table 1:** Yield, total soluble solids (TSS), water activity (a_w_), and surface color parameters (L*, a*, and b*) of dried cocoa beans fermented at controlled temperatures of 15, 25, 35, and 45 °C.

Temperature (°C)	Yield (%)	Total Soluble Solids (°Brix)	L* Value	a* Value	b* Value	a_w_
15	47.17 ± 0.76 ^a^	3.27 ± 0.15 ^a^	47.44 ± 5.73 ^a^	15.66 ± 3.56	22.16 ± 9.10	0.57 ± 0.01 ^a^
25	45.00 ± 0.50 ^b^	3.07 ± 0.15 ^a^	41.79 ± 1.46 ^ab^	16.04 ± 1.19	19.12 ± 1.68	0.49 ± 0.02 ^b^
35	42.67 ± 0.29 ^c^	2.80 ± 0.36 ^ab^	34.85 ± 3.20 ^b^	14.57 ± 2.81	15.00 ± 5.49	0.49 ± 0.04 ^b^
45	37.67 ± 0.76 ^d^	2.23 ± 0.12 ^b^	41.27 ± 0.96 ^ab^	19.44 ± 0.92	20.61 ± 1.69	0.46 ± 0.04 ^b^

Values are mean ± standard deviation. Different letters indicate significant differences at *p* < 0.05.

**Table 2:** Moisture content, total titratable acidity, reducing sugar content, total phenolic content, theobromine and catechin content of dried cocoa beans fermented at controlled temperatures of 15, 25, 35, and 45 °C.

Temperature (°C)	Moisture Content (%)	Total Titratable Acidity (% db)	Reducing Sugar (mg/g db)	Total Phenolic Compound (mg GAE/g db)	Theobromine (mg/g db)	Catechin (mg/g db)
15	6.26 ± 0.21 ^a^	0.26 ± 0.02 ^b^	17.84 ± 2.82 ^a^	2.39 ± 0.37 ^a^	3.62 ± 0.53	0.03 ± 0.02 ^b^
25	5.51 ± 0.29 ^a^	0.35 ± 0.02 ^a^	4.79 ± 0.53 ^b^	1.38 ± 0.07 ^b^	3.38 ± 0.39	0.07 ± 0.02 ^ab^
35	4.55 ± 0.40 ^b^	0.35 ± 0.02 ^a^	5.28 ± 0.21 ^b^	1.45 ± 0.24 ^b^	3.87 ± 0.18	0.09 ± 0.02 ^a^
45	4.11 ± 0.41 ^b^	0.39 ± 0.02 ^a^	3.24 ± 0.44 ^b^	0.36 ± 0.17 ^c^	3.85 ± 0.18	0.10 ± 0.02 ^a^

Values are mean ± standard deviation. Different letters indicate significant differences at *p* < 0.05. db: dry basis.

## Data Availability

The data presented in this study are available from the corresponding author upon reasonable request.

## Supplementary Materials

The following supporting information can be downloaded at: https://www.mdpi.com/article/10.3390/foods15193473/s1, Figure S1: Moisture content during fermentation; Figure S2: Measure temperature during fermentation; Figure S3: Reducing sugar content during fermentation; Figure S4: Total soluble solid during fermentation; Figure S5: pH during fermentation; Figure S6: Total titratable acidity during fermentation; Figure S7: Total phenolic content during fermentation; Figure S8: *L***a***b** color on surface during fermentation; Figure S9: *L***a***b** color on cotyledons during fermentation; Figure S10: Total sugar content during fermentation; Figure S11: Ethanol content during fermentation; Figure S12: Lactic acid content during fermentation; Figure S13: Acetic acid content during fermentation; Table S1: Changes in internal cotyledon color parameters, pH, and total titratable acidity between fermentation days 6 and 8.
